# Cyclin F/FBXO1 Interacts with HIV-1 Viral Infectivity Factor (Vif) and Restricts Progeny Virion Infectivity by Ubiquitination and Proteasomal Degradation of Vif Protein through SCF^cyclin F^ E3 Ligase Machinery*[Fn FN2]

**DOI:** 10.1074/jbc.M116.765842

**Published:** 2017-02-09

**Authors:** Tracy Augustine, Priyanka Chaudhary, Kailash Gupta, Sehbanul Islam, Payel Ghosh, Manas Kumar Santra, Debashis Mitra

**Affiliations:** From the ‡ National Centre for Cell Science, Pune, Maharashtra 411007, India and; the § Bioinformatics Centre, Savitribai Phule Pune University, Pune, Maharashtra 411007, India

**Keywords:** E3 ubiquitin ligase, host-pathogen interaction, human immunodeficiency virus (HIV), protein degradation, ubiquitylation (ubiquitination), APOBEC3G, cyclin F, F-box protein, Vif, Virion infectivity

## Abstract

Cyclin F protein, also known as FBXO1, is the largest among all cyclins and oscillates in the cell cycle like other cyclins. Apart from being a G_2_/M cyclin, cyclin F functions as the substrate-binding subunit of SCF^cyclin F^ E3 ubiquitin ligase. In a gene expression analysis performed to identify novel gene modulations associated with cell cycle dysregulation during HIV-1 infection in CD4^+^ T cells, we observed down-regulation of the cyclin F gene (*CCNF*). Later, using gene overexpression and knockdown studies, we identified cyclin F as negatively influencing HIV-1 viral infectivity without any significant impact on virus production. Subsequently, we found that cyclin F negatively regulates the expression of viral protein Vif (viral infectivity factor) at the protein level. We also identified a novel host-pathogen interaction between cyclin F and Vif protein in T cells during HIV-1 infection. Mutational analysis of a cyclin F-specific amino acid motif in the C-terminal region of Vif indicated rescue of the protein from cyclin F-mediated down-regulation. Subsequently, we showed that Vif is a novel substrate of the SCF^cyclin F^ E3 ligase, where cyclin F mediates the ubiquitination and proteasomal degradation of Vif through physical interaction. Finally, we showed that cyclin F augments APOBEC3G expression through degradation of Vif to regulate infectivity of progeny virions. Taken together, our results demonstrate that cyclin F is a novel F-box protein that functions as an intrinsic cellular regulator of HIV-1 Vif and has a negative regulatory effect on the maintenance of viral infectivity by restoring APOBEC3G expression.

## Introduction

HIV-1 has devised numerous mechanisms to evade the host immune system and establish itself successfully within the host. To accomplish this, HIV-1 exploits a multitude of cellular host factors and even mimics some of their functions. The host system in turn employs numerous competitive strategies to inhibit the invasion of virus for its self-protection. A productive infection, hence, is the outcome of continuous conflicts between the host and the virus where the latter is able to outrun the host defense.

Restriction factors constitute an expanding group of cellular proteins that create powerful barriers to the virus, the most well characterized of them being the APOBEC3 family of proteins (1, 2), TRIM5 (3, 4), BST2/tetherin (5, 6), SAMHD1 (7, 8), Mx2/MxB (9, 10), and the recently identified SERINC3 and SERINC5 (11, 12). HIV-1 impedes these restrictive mechanisms of the host, mostly with the help of its highly evolved accessory proteins, Nef, Vif, Vpr, and Vpu, by using them to either exploit or oppose the functions of various host cellular factors.

HIV-1 viral infectivity factor (Vif),^2^ one of the accessory proteins, is responsible for the infectivity of the virus. The well established function of HIV-1 Vif is the ubiquitination and subsequent proteasomal degradation of host restriction factor APOBEC3G (2, 13) and to different extents other APOBEC3 family members (14). APOBEC3G is a cytidine deaminase, which incorporates into progeny virions of retroviruses and inhibits viral replication by inducing G to A hypermutations in the plus strand of viral DNA. HIV-1 Vif hijacks the ubiquitin proteasome system comprising Cul5, EloB, and EloC and recruits transcription factor CBFβ (15, 16) to mediate the ubiquitination and proteasomal degradation of APOBEC3G. Therefore, approaches that disrupt the Vif-APOBEC3G interaction can help to restore APOBEC3G levels, hence reducing virion infectivity. Importantly, HIV-1 Vif has also previously been reported to be ubiquitinated (17) and proteasomally degraded (18). However, a clear picture of Vif ubiquitination and its proteasomal degradation during HIV-1 infection has so far remained elusive.

Of late, there have been reports of the significance of certain cellular factors associated with the cell cycle in HIV-1 pathogenesis. Cyclin T1, as well as its CDK partner CDK9, is among the foremost cyclin-CDK complexes involved in HIV-1 transcription regulation, as they form a part of the PTEF-B complex and act as a cofactor for HIV-1 Tat-induced transcription elongation of the viral genome (19, 20). On the contrary, cyclin K acts as a competitor of the cyclin T1-CDK9 complex, as it binds to CDK9 and restricts its nuclear translocation in a Nef-dependent manner, thereby inhibiting HIV-1 gene expression and replication (21). Other cell cycle-associated genes identified as playing significant role in HIV-1 infection include p21/CDKN1A, which acts as an intrinsic inhibitor of HIV-1 reverse transcription in CD4^+^ T cells of elite controllers (22); and cyclin A2 (23) and cyclin L2 (24), which are exploited by HIV-1 to evade the restriction function of SAMHD1 in different cell types, thereby enabling the virus to replicate efficiently.

Cyclin F is a unique member of the cyclin family of proteins, as it does not bind or activate any CDKs, unlike typical cyclins (25–27). However, cyclin F oscillates in the cell cycle, with an expression peak at G_2_ (28, 29). Cyclin F is also the pioneering member of the F-box family of proteins owing to the identification of the F-box domain in it. F-box proteins are the substrate-binding subunits of the SCF (Skp1-Cullin 1-F-box) E3 ligases (30). Cyclin F, unlike other cyclins, utilizes its cyclin domain to bind to specific substrates signaled for degradation (28, 29). Cyclin F has been identified as involved in cell cycle regulation by timely degradation of the centrosomal protein CP110 (29), microtubule-associated protein NuSAP (31), and cell division cycle 6 (CDC6) protein (32); cyclin F also maintains a balanced cellular dNTP level by binding and degrading RRM2 (28). Cyclin F has also been shown to suppress B-Myb activity to ensure G_2_ checkpoint control in response to IR-induced DNA damage (33). These cellular regulatory functions mediated by cyclin F have enabled researchers to consider cyclin F as a potential molecular target in cancer therapeutics. However, there has been no evidence thus far for any role of cyclin F in the context of HIV-1 infection.

In the present work, we performed HIV-1-induced cell cycle-associated gene expression analysis in infected primary CD4^+^ T cells isolated from PBMCs of seronegative donors. Cyclin F was identified from the gene expression analysis as a significantly down-regulated gene during infection. Further investigation of the role of cyclin F in infection revealed its negative regulatory influence on HIV-1 viral infectivity. Subsequent analysis elucidated a novel physical interaction between cyclin F and viral protein Vif by which cyclin F regulates Vif expression in a proteasome-dependent manner, correlating with a direct implication of HIV-1 progeny virion infectivity.

## Results

### 

#### 

##### Cell Cycle-associated Gene Expression Analysis in CD4^+^ T Cells Identifies Cyclin F Down-regulation during HIV-1 Infection

HIV-1 infection induces cell cycle arrest at the G_2_/M phase, as it is essential for optimal expression of the viral genome. The HIV-1 long terminal repeat (LTR) promoter is reported to be most active in this phase (34). G_2_/M phase arrest is brought about by two viral proteins, Vpr (34, 35) and Vif (36). Apart from induction of G_2_/M arrest in cells, some cell cycle-associated genes have also been identified as playing a crucial role in HIV-1 pathogenesis (19, 23, 24). Hence, we decided to specifically analyze cell cycle-associated gene modulations during HIV-1 infection in CD4^+^ T cells to identify novel host-pathogen interactions in this context.

**FIGURE 1.**
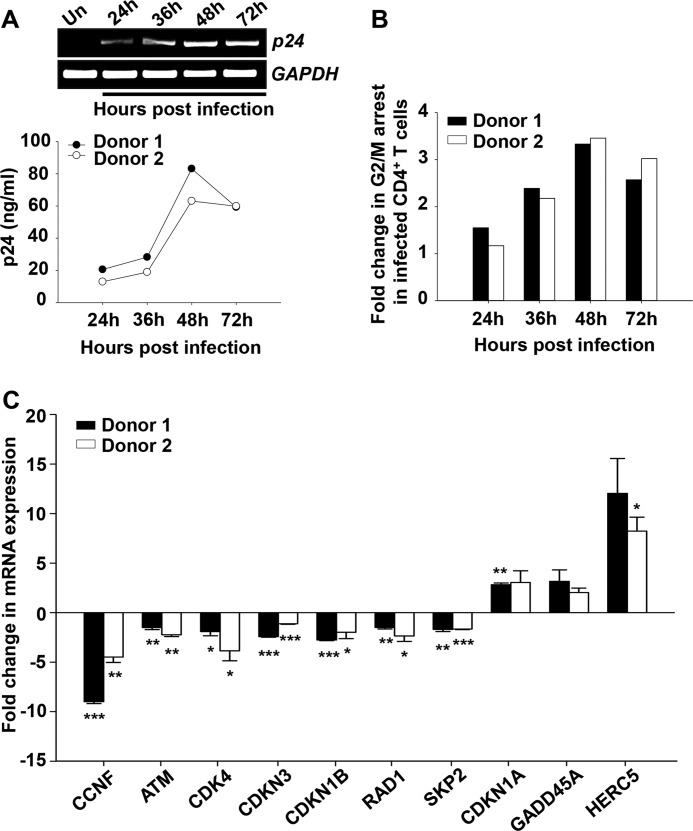
**HIV-1 NL4-3 infection in human CD4^+^ T cells and validation of results for selected differentially expressed cell cycle associated genes observed in PCR array.** *A*, kinetic expression profile of p24 in HIV-1-infected CD4^+^ T cells at mRNA level (*upper panel*) and in culture supernatants (*lower panel*) as analyzed by RT-PCR and p24 antigen capture ELISA, respectively. *Un*, uninfected. *B*, -fold change in G_2_/M arrest in HIV-1-infected CD4^+^ T cells as compared with uninfected cells. *C*, validation of selected genes identified as modulated from cell cycle PCR array results. Shown is the -fold change in the expression pattern of selected cell cycle-associated genes in HIV-1-infected CD4^+^ T cells compared with uninfected CD4^+^ T cells, as analyzed by quantitative real-time RT-PCR. Data represent mean ± S.E.

To carry out this objective, we performed gene expression profiling using a real time-based cell cycle PCR array containing primers of 84 cell cycle pathway-associated genes. CD4^+^ T cells were isolated from PBMCs obtained from seronegative donors. These cells were activated using PHA/IL-2 and were infected using HIV-1 NL4-3 (0.5 m.o.i.) virus. Infection progression was monitored using a p24 antigen capture ELISA. We observed that the infection peaked at 48 h post-infection (hpi) (Fig. 1*A*). Cell cycle progression monitored using propidium iodide staining along the same time points showed significant G_2_/M arrest at 48 hpi (Fig. 1*B*). On the basis of the above time kinetics, cDNA from cells harvested 48 hpi was used to perform gene expression analysis. Uninfected cDNA from the same time point served as control. From the results obtained, a comparison of gene expression analysis identified several genes as modulated during HIV-1 infection. Genes that showed more than 2-fold modulation are listed in supplemental Table 1. The modulated genes are also listed on the basis of the cell cycle phases in which they are prominently present (Table 1). We further validated the expression of some of the most prominently modulated genes from different cell cycle phases in CD4^+^ T cells from two seronegative donors post-infection (Fig. 1*C*).

**TABLE 1:** **Selected genes in the PCR array modulated during HIV-1 infection and classified on the basis of cell cycle phases** This is a list of cell cycle-associated genes that showed more than 2-fold modulation in primary CD4^+^ T cells with HIV-1 NL4–3 infection. The -fold change values and NM_ID are detailed in supplemental Table 1.

Cell cycle phase	Genes showing >2-fold modulation
G_1_ and G_1_-S phase	*ANAPC2, CCNE1, CDC34, CDK4, CDK6, CDKN1B, CDKN3, CUL3, SKP2*
G_2_ phase and G_2_/M transition	*ANAPC2, BCCIP, CCNB1, CCNT1, CDKN3, CKS2, DNM2, GTSE1, HERC5, KPNA2*
M phase	*CCNB2, CCNF, CDC16, CDC20*
S phase and DNA replication	*MCM2, MCM3, MCM4, MCM5*
Cell cycle check point and cell cycle arrest	*ATM, BRCA1, CCNG2, CDC34, CDKN1A, CDKN1B, CDKN2A, CDKN2B, CDKN3, CUL3, GADD45A, HUS1, KNTC1, MAD2L1, RAD1, TP53*
Regulation of cell cycle	*ANAPC2, ATM, BCCIP, CCNB1, CCNB2, CCNE1, CCNF, CCNT1, CDC16, CDC20, CDK4, CDK6, CDKN1A, CDKN1B, GADD45A, KNTC1, MK167, SKP2, TFDP1*
Negative regulation of cell cycle	*ATM, BRCA1, CDKN2B, RBL1, TP53*

**FIGURE 2.**
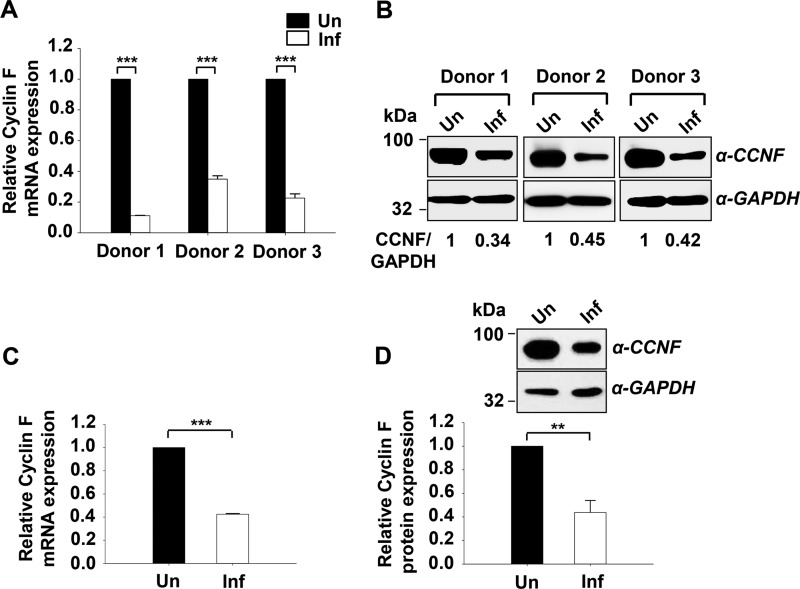
**Cyclin F is down-regulated in HIV-1-infected CD4^+^ T cells.** *A*, cyclin F undergoes down-regulation in CD4^+^ T cells isolated from healthy donors during HIV-1 infection at mRNA level (48 hpi). CD4^+^ T cells isolated from PBMCs of healthy donors obtained from a local blood bank were activated with PHA (5 μg/ml) followed by 0.5 m.o.i. infection in the presence of IL-2 (20 units/ml). *B*, down-regulation of cyclin F at protein level (72 hpi) in HIV-1-infected CD4^+^ T cells as analyzed by immunoblotting. *C*, cyclin F undergoes down-regulation with HIV-1 infection (0.5 m.o.i.) in Jurkat T cells (48 hpi) as analyzed by quantitative RT-PCR. *D*, down-regulation of cyclin F at the protein level (72 hpi) in HIV-1-infected Jurkat cells (*upper panel*). Densitometric analysis for the same (*n* = 3) is shown in the *lower panel. Un*, uninfected; *Inf*, infected. Data represent mean ± S.E.

The cyclin F gene (*CCNF*) displayed the highest down-regulation at the mRNA level among the genes that showed significant modulation. Further expression analysis of cyclin F at mRNA (Fig. 2*A*) and protein (Fig. 2*B*) levels in infected CD4^+^ T cells confirmed its significant down-regulation with infection. We also observed cyclin F down-regulation in infected Jurkat cells (human CD4^+^ T cell line) at both the mRNA (Fig. 2*C*) and protein levels (Fig. 2*D*). Similar down-regulation was also obtained in another CD4^+^ T cell line, CEM-GFP, the results for which are shown in Fig. 4*A*. These observations led us to further characterize the potential implication of cyclin F down-regulation in HIV-1 infection.

##### Cyclin F Negatively Regulates the Infectivity of Progeny Virions

**FIGURE 3.**
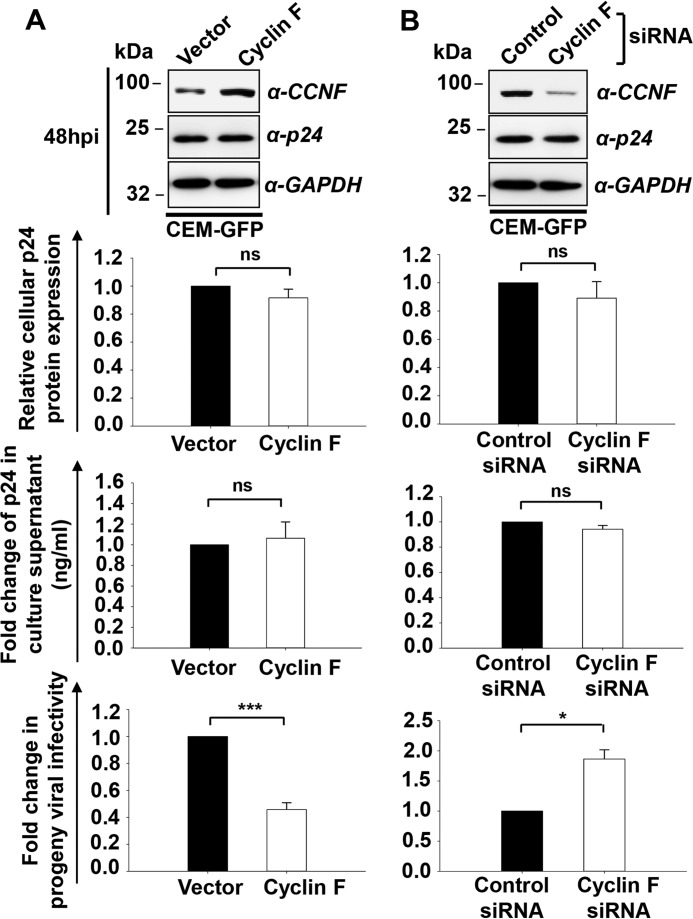
**Cyclin F interferes with infectivity of progeny virions in HIV-1 NL4-3-infected CEM-GFP T cells.** *A*, cyclin F overexpression does not influence p24 gene expression or viral production, but it reduces the infectivity of progeny virions. Cyclin F was overexpressed in CEM-GFP T cells infected with HIV-1 NL4-3 virus (0.5 m.o.i.) 24 h post-transfection. Virus production was compared using p24 ELISA of culture supernatants (48 hpi) (*n* = 3), and these supernatants were used for a comparison of progeny virion infectivity using TZM-bl reporter cells by β-gal staining (*n* = 3). *B*, silencing of cyclin F expression in CEM-GFP cells enhances progeny viral infectivity. Cyclin F was silenced in CEM-GFP cells using cyclin F siRNA (200 nm), and cells were infected (0.5 m.o.i.) 24 h post-transfection. Quantitation of the -fold change in infectivity of progeny virions is shown as described in *A* (*n* = 3). Data represent mean ± S.E.

The down-regulation of cyclin F expression in HIV-1 infection led us to speculate that cyclin F could have a possible role in viral pathogenesis. As cyclin F undergoes down-regulation during infection, to understand its role in HIV-1 pathogenesis, we overexpressed cyclin F in CEM-GFP T cells. After 24 h of transfection, the cells were infected with 0.5 m.o.i. HIV-1 NL4-3 virus. Cells were harvested 48 hpi, and immunoblotting for p24 gag protein showed no significant difference in the relative cellular expression levels of p24 (Fig. 3*A*, *top*). The cell culture supernatants collected and used to compare virus production using p24 antigen capture ELISA also did not show significant differences in the production of virions with cyclin F overexpression (Fig. 3*A*, *middle*). Further, the viral supernatants were normalized and were used to compare viral infectivity using a TZM-bl indicator assay as described under “Experimental Procedures.” Here, surprisingly, we observed a significant decrease in progeny virion infectivity of viral supernatants from cyclin F-overexpressed conditions (Fig. 3*A*, *bottom*).

To confirm this observation, endogenous cyclin F was silenced using siRNA pool (GE Healthcare Dharmacon) against cyclin F in CEM-GFP cells. Non-targeting control siRNA pool served as the control. After 24 h of transfection, cells were infected with 0.5 m.o.i. NL4-3 virus. Cells were harvested 48 hpi, and gene silencing was confirmed by immunoblotting using cyclin F antibody. Further we analyzed the expression of p24 using immunoblotting, and the culture supernatants collected were used to detect virus production as well as perform viral infectivity assays after normalization. In agreement with the overexpression results, we did not observe any significant variations in cellular p24 expression (Fig. 3*B*, *top*) and production of HIV-1 virions (Fig. 3*B*, *middle*). On comparison of viral infectivity of supernatants from control and cyclin F-silenced cells, we observed a significant enhancement in viral infectivity of virions produced by silencing cyclin F (Fig. 3*B*, *bottom*). The above results therefore point to an important functional implication of cyclin F in regulating the infectivity of virions produced during HIV-1 infection.

##### Cyclin F Regulates HIV-1 Vif Expression during Infection

**FIGURE 4.**
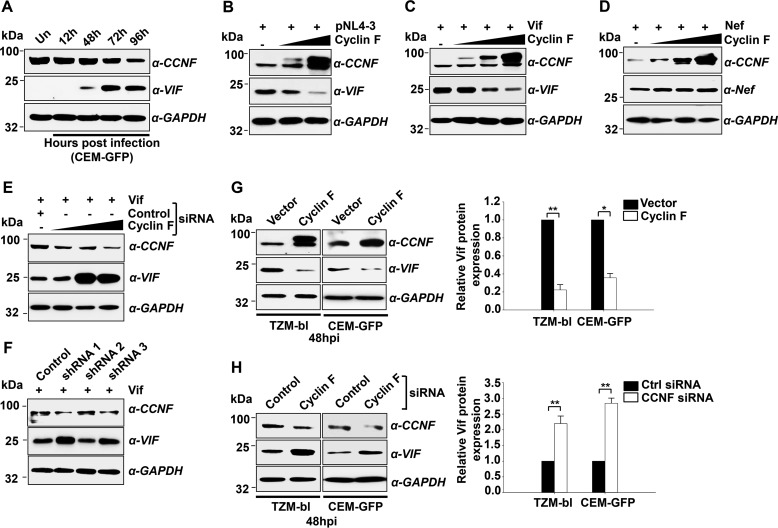
**Cyclin F impairs the cellular expression of HIV-1 Vif.** *A*, expression analysis of cyclin F and Vif in HIV-1-infected (0.5 m.o.i.) CEM-GFP T cells as analyzed by immunoblotting. *Un*, uninfected. *B*, cyclin F overexpression decreases Vif expression in a dose-dependent manner in HIV-1 pNL4-3-transfected HEK293T cells. *C*, Vif down-regulation by cyclin F is independent of the presence of other viral proteins. HEK293T cells were transfected with increasing concentrations of cyclin F along with Vif followed by immunoblotting of the lysates. *D*, cyclin F does not regulate the expression of HIV-1 Nef protein. HEK293T cells were transfected with increasing concentrations of cyclin F along with Nef followed by immunoblotting of the lysates. *E*, siRNA-mediated knockdown of cyclin F increases Vif expression. HEK293T cells were transfected with Vif expression vector and increasing concentrations of cyclin F siRNA (50, 100, and 200 nm) followed by immunoblotting for Vif. *F*, knockdown of cyclin F using shRNA increases Vif expression. HEK293T cells were transfected with Vif expression vector and three different cyclin F shRNA constructs. Cells harvested at 72 h post-transfection were used for immunoblotting. All *panels* are representatives of at least three independent experiments. *G*, cyclin F down-regulates Vif expression during HIV-1 infection. Cyclin F was overexpressed in TZM-bl and CEM-GFP cells and the cells were infected with HIV-1 NL4-3 virus (0.5 m.o.i.) 24 h post-transfection. Cells were harvested 48 hpi, and the lysates were immunoblotted for Vif. Densitometric analysis for the same is shown (*n* = 3). *H*, cyclin F silencing increases Vif expression during HIV-1 infection. Cyclin F siRNA (200 nm) transfection was followed by 0.5 m.o.i. infection in TZM-bl and CEM-GFP cells. Cells were harvested 48 hpi and analyzed for Vif expression by immunoblotting. Densitometric analysis for the same is shown (*n* = 3). Data represent mean ± S.E.

As HIV-1 Vif is known to be intimately associated with viral infectivity, we speculated that cyclin F would have a possible effect on Vif expression. The expression profile of cyclin F and Vif at the protein level was simultaneously analyzed in HIV-infected CEM-GFP cells. Cyclin F, as expected, showed a progressive down-regulation, whereas Vif, a late expressing viral protein, increased and stabilized its expression with the progression of infection (Fig. 4*A*). To further analyze this finding, HEK293T cells were co-transfected with increasing concentrations of pCMV-FLAG-cyclin F along with pNL4-3. Protein expression analysis showed a significant and dose-dependent reduction in Vif expression with cyclin F overexpression (Fig. 4*B*). To confirm the inverse correlation of cyclin F and Vif expression, we co-transfected Vif expression construct along with increasing doses of cyclin F in HEK293T cells. Here again, we observed a gradual loss of Vif expression with increasing amounts of cyclin F, indicating that cyclin F-mediated Vif down-regulation is direct and is independent of the presence of other viral proteins (Fig. 4*C*). To confirm the specificity of cyclin F in Vif regulation, HEK293T cells were then co-transfected with increasing doses of cyclin F along with HIV-1 Nef, another viral protein known to be important for viral infectivity (12, 37). However, we did not observe any significant impact of cyclin F overexpression on HIV-1 Nef expression (Fig. 4*D*). The regulatory effect of cyclin F on Vif was further confirmed by cyclin F knockdown in HEK293T cells, where dose-dependent siRNA-mediated silencing of cyclin F led to a simultaneous increase in Vif expression (Fig. 4*E*). Additionally, we also performed a transient transfection of three different lentiviral shRNA constructs of cyclin F along with Vif in HEK293T cells to analyze the effect on Vif expression. Cells harvested 72 h post-transfection showed silencing of cyclin F by two among the three shRNA constructs (shRNA1 and shRNA3) with simultaneous stabilization of Vif expression as compared with control (Fig. 4*F*), further strengthening the negative regulation of Vif by cyclin F. The sequences and clone IDs of the shRNA used are given under “Experimental Procedures.” Further, to understand the significance of cyclin F in the context of HIV-1 infection, we performed overexpression and knockdown of cyclin F in TZM-bl as well as CEM-GFP cells followed by 0.5 m.o.i. HIV-1 infection. Vif expression was found to be down-modulated with the overexpression of cyclin F and up-regulated with the silencing of cyclin F during HIV-1 infection in both TZM-bl and CEM-GFP cells (Fig. 4, *G* and *H*). Interestingly, we also observed that cyclin F expression did not alter Vif mRNA expression (as shown in Fig. 7*A*). These results thus substantiate that cyclin F negatively regulates the cellular expression of HIV-1 Vif protein during infection.

##### Cyclin F Physically Interacts and Co-localizes with HIV-1 Vif during Infection

**FIGURE 5.**
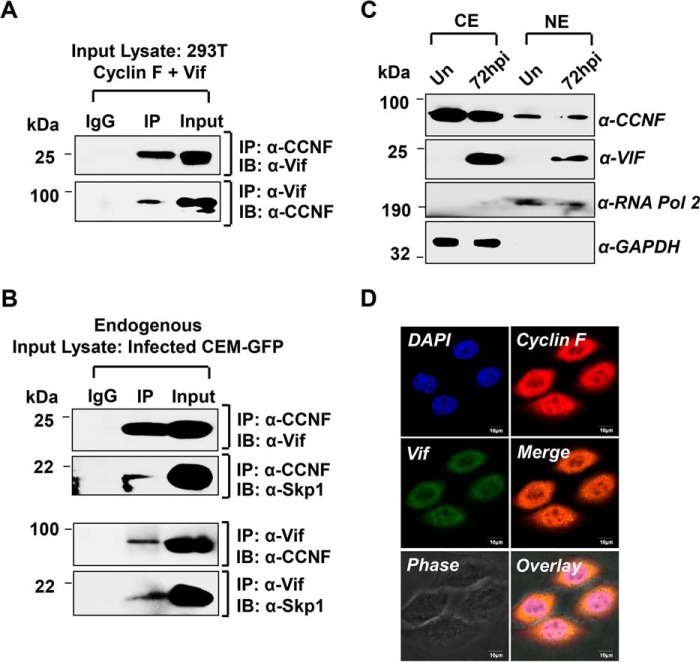
**Cyclin F physically interacts with Vif during HIV-1 infection in T cells.** *A*, co-immunoprecipitation and reverse co-immunoprecipitation showing interaction of cyclin F and HIV-1 Vif in transfected HEK293T cells. *B*, cyclin F and Vif interacts endogenously during HIV-1 infection in 0.5 m.o.i. infected CEM-GFP (72 hpi) cells. Skp1 is also co-immunoprecipitated with cyclin F and Vif pulldown. *C*, cyclin F and Vif is present in the nucleus (*NE*) as well as the cytoplasm (*CE*) during infection. *D*, co-localization of cyclin F and Vif in both nuclear and cytoplasmic compartments in HIV-1-infected TZM-bl cells observed at 24 h post-infection. All *panels* are representative of at least three independent experiments. *IP*, immunoprecipitation; *IB*, immunoblot.

To explore the possibility of a cyclin F-Vif association, expression constructs of both proteins were co-transfected in HEK293T cells, and lysates were harvested 48 h post-transfection were used for co-immunoprecipitation assays. Immunoprecipitation using cyclin F antibody followed by immunoblotting using Vif antibody detected Vif in the immunoprecipitated sample (Fig. 5*A*, *upper panel*). Reverse co-immunoprecipitation assay using Vif antibody for pulldown and subsequent immunoblotting with cyclin F antibody substantiated the interaction between the two proteins (Fig. 5*A*, *lower panel*). The interaction was further confirmed similarly under endogenous conditions by means of co-IP and reverse co-IP using cyclin F and Vif antibodies in HIV-1-infected (0.5 m.o.i.) CEM-GFP cells harvested 72 h post-infection (Fig. 5*B*). Co-IP analysis of Skp1 with cyclin F as well as Vif pulldown served as a positive control for the presence of the intact SCF^cyclin F^ complex in the infected lysates (Fig. 5*B*). As Skp1 links F-box protein cyclin F to the SCF complex and thereby to the ubiquitin machinery (25), the above results indicate that cyclin F could possibly regulate HIV-1 Vif through the ubiquitin machinery involving SCF^cyclin F^ E3 ligase.

Cyclin F, although reported to be functionally relevant in the cell nucleus as well as the centrosome (28, 29), is also found in the perinuclear region (30) and the cytoplasm (38). On the contrary, Vif is functionally important in the cytoplasm, where it degrades APOBEC3G, although a substantial amount of it has been detected in the nucleus as well (39, 40). Subcellular compartmentalization of cyclin F and Vif was therefore analyzed in 0.5 m.o.i. infected CEM-GFP cells harvested at 72 hpi. Cytoplasmic and nuclear extracts were prepared and analyzed, and they showed the presence of cyclin F and Vif in both the cytoplasm as well as the nucleus during infection (Fig. 5*C*). Immunostaining of cyclin F and Vif performed in HIV-1-infected TZM-bl cells showed co-localization of both the proteins in nuclear as well as cytoplasmic compartments (Fig. 5*D*).

##### Molecular Docking Analysis of Cyclin F and HIV-1 Vif Interaction

It is well established that cyclins bind to their specific CDKs/substrates through their cyclin domain. A hydrophobic patch of amino acids, MRYIL, in the cyclin domain of cyclin F binds to putative the “CY” motif, R*X*L/R*X*I, on its substrates (28, 32, 33). To gain clarity in the cyclin F-Vif interaction, we scanned the amino acid sequence of HIV-1 NL4-3 Vif, which revealed the presence of a single CY motif (RKL) at position 167–169 in the C-terminal region of Vif. We hypothesized this motif as the specific cyclin F-binding region on Vif. To confirm this hypothesis, docking studies were performed between the known Vif structure and a modeled structure of cyclin F protein using the PatchDock server.

**FIGURE 6.**
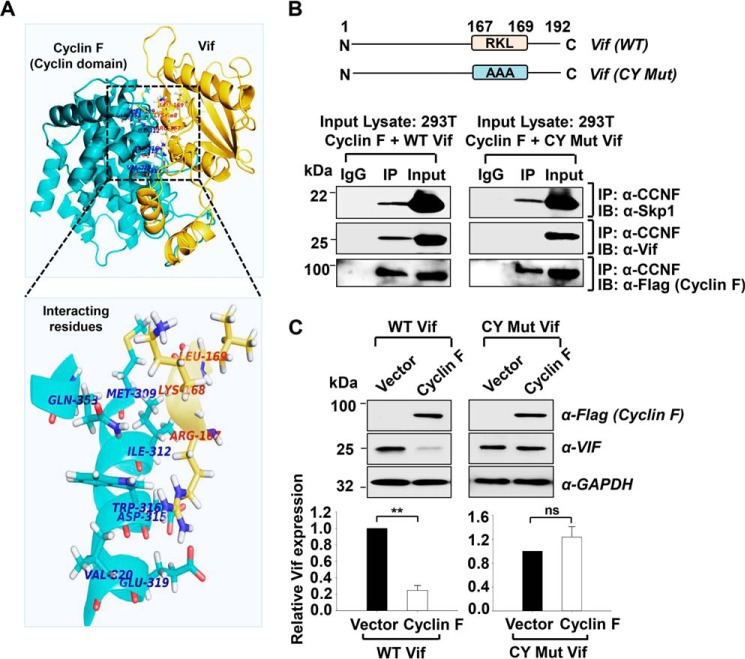
**Cyclin F binds to Vif through the CY motif in the C-terminal region of HIV-1 NL4-3 Vif.** *A*, molecular docking of modeled cyclin domain region (including cyclin C domain) of cyclin F with available HIV-1 Vif structure. Amino acid residues in cyclin F and Vif involved in the interaction as predicted by PatchDock Server are shown in the *zoomed image below. B*, depiction of CY mutant generation of Vif (*upper panel*). Cyclin F does not co-immunoprecipitate CY mutant Vif (*lower panel*). Cyclin F-Skp1 interaction is found to be intact, but cyclin F-Vif interaction is lost when CY mutant of Vif is co-transfected with cyclin F in transfected 293T cells. All *panels* represent data from at least two or more independent experiments. *IP*, immunoprecipitate; *IB*, immunoblot. *C*, CY mutant Vif is not down-regulated by cyclin F in transfected 293T cells (*n* = 2). Data represent mean ± S.E.

Analysis of the docking models showed an interaction between the C-terminal region of Vif (^167^RKL^169^) with several binding sites in the cyclin domain of cyclin F (Fig. 6*A*, *top*). The intermolecular protein-protein interactions and surface interface areas of the docked complexes were determined using the PISA server. The selected docked complex of cyclin F-Vif showed an interaction having an interface area of 1745.1 Å^2^ and solvation free energy (Δ*^i^G*) of −10.4 kcal/mol. Docking analysis showed that Arg-167 and Lys-168 of the Vif protein are involved in cyclin F-Vif binding, which involves cyclin F-specific residues Met-309, Ile-312, Asp-315, Trp-316, Glu-319, Thr-322, Met-323, Leu-352, Gln-353, Leu-380, and Thr-381 (Fig. 6*A*, below). The docking study thus suggests a crucial role for Arg-167 and Lys-168 residues of the Vif protein in retaining the interactions with cyclin F protein.

##### Cyclin F Binds to Vif through the CY Motif (RXL) in the C-terminal Region of HIV-1 Vif

To analyze the bioinformatics-based predictions of the cyclin F-Vif interaction, as well as on the basis of previous reports on cyclin F-interacting amino acid motif, a Vif point mutant (RKL/AAA-CY Mut Vif) was constructed (Fig. 6*B*) at amino acid residues 167–169 using site-directed mutagenesis. The generation of the point mutant was confirmed by nucleotide sequence analysis. This CY Mut Vif was further used for co-immunoprecipitation assays with cyclin F. Co-transfection of HEK293T cells with the wild-type or CY Mut Vif along with cyclin F was followed by co-immunoprecipitation analysis at 48 h post-transfection. Lysates were pulled down using cyclin F antibody followed by probing with Vif antibody. Interestingly, we observed that cyclin F could not pull down the CY Mut Vif along with it (Fig. 6*B*). As a positive control, co-IP of cyclin F with Skp1 was also analyzed, which showed an interaction of both in wild-type as well as CY Mut-transfected cells (Fig. 6*B*). Positive immunoprecipitation for cyclin F is also shown, as detected by anti-FLAG antibody (Fig. 6*B*). Further, the expression of wild-type and mutant Vif was analyzed in the presence of overexpressed cyclin F. As seen previously, the expression of wild-type Vif decreased as a result of cyclin F overexpression, but the CY Mut Vif showed intact expression with respect to the control (Fig. 6*C*). These experiments suggest that cyclin F interaction occurs at the CY motif (^167^Arg-Lys-Leu^169^) on HIV-1 NL4-3 Vif and that this interaction is essential for cyclin F-mediated regulation of Vif expression.

##### SCF^cyclin F^ E3 Ligase Induces Ubiquitination and Proteasomal Degradation of HIV-1 Vif during Infection

**FIGURE 7.**
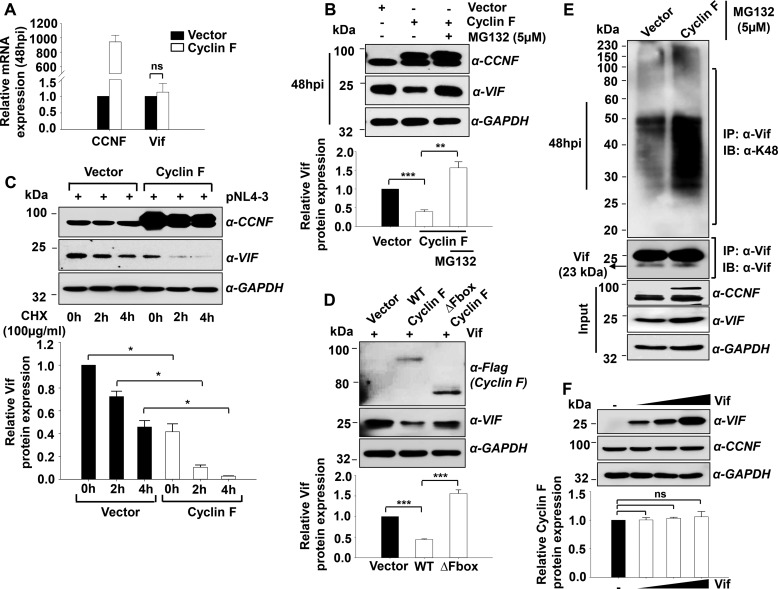
**Cyclin F ubiquitinates and proteasomally degrades Vif during HIV-1 infection.** *A*, cyclin F overexpression does not affect Vif mRNA expression during HIV-1 infection. Cyclin F was overexpressed in TZM-bl, and cells were infected (0.5 m.o.i.) 24 h post-transfection for analysis of Vif expression at the mRNA level (*n* = 3) 48 h post-infection. *B*, MG132 rescues proteasomal degradation of Vif in cyclin F overexpressed in HIV-1-infected cells. Cyclin F was overexpressed in TZM-bl cells, and cells were infected (0.5 m.o.i.) 24 h post-transfection. The cells show restoration of Vif expression with the addition of MG132 (*upper panel*) after 48 h post-infection. Densitometric analysis for the same is shown in the *lower panel* (*n* = 3). *C*, cycloheximide (*CHX*, 100 μg/ml) pulse chase also revealed accelerated turnover kinetics of Vif with cyclin F overexpression (*upper panel*). Densitometric analysis for the same is shown in the *lower panel* (*n* = 2). *D*, cyclin F deletion mutant, ΔFbox cyclin F, is unable to degrade Vif (*upper panel*). Densitometric analysis for the same is shown in the *lower panel* (*n* = 3) *E*, cyclin F polyubiquitinates Vif during HIV-1 infection. TZM-bl cells overexpressed with cyclin F and subsequently infected with NL4-3 virus showed enhanced laddering, compared with vector control, when immunoprecipitated (*IP*) with Vif antibody and probed using Lys-48 ubiquitin linkage-specific antibody. MG132 was added to vector control as well as cyclin F-overexpressed cells 12 h prior to harvesting. All *panels* represent data from at least two or more independent experiments. *IB*, immunoblot. *F*, Vif overexpression does not alter cyclin F expression, indicating that Vif may not have a direct impact on cyclin F down-regulation during HIV-1 infection (*n* = 2). Data represent mean ± S.E.

As cyclin F is the substrate-binding subunit of the SCF^cyclin F^ E3 ligase, which interacts with its specific substrates and directs them for proteasomal degradation, we hypothesized that the same mechanism could be involved in the cyclin F-mediated down-regulation of HIV-1 Vif expression. To scrutinize this possibility, we first analyzed the mRNA level expression changes of HIV-1 Vif with cyclin F overexpression. TZM-bl cells were used here, as they can be efficiently transfected and also can be infected. Cyclin F was overexpressed in these cells followed by 0.5 m.o.i. infection and were analyzed for Vif expression at the mRNA level, which did not show any significant differences between the vector control and cyclin F overexpressed conditions (Fig. 7*A*), indicating that cyclin F-mediated regulation of Vif expression could be post-translational.

Next we analyzed the effect of proteasomal inhibitor MG132 on Vif expression in infected TZM-bl cells in the presence of cyclin F. MG132 (5 μm) was added to the media 12 h prior to harvesting the infected cells. Complete restoration of Vif expression was observed with the addition of MG132 in the cyclin F overexpressed condition (Fig. 7*B*), demonstrating that cyclin F mediates proteasomal degradation of Vif during infection. This was further validated by a cycloheximide pulse-chase assay in HEK293T cells, where we found that the turnover kinetics of Vif was accelerated upon overexpression of cyclin F (Fig. 7*C*).

Cyclin F forms a complex with SCF machinery through Skp1 interaction via its F-box domain (25). To confirm the above observations, we constructed an F-box deletion mutant of cyclin F, pCMV-FLAG-ΔFbox-cyclin F, and used it for co-transfection experiments along with a Vif expression construct in HEK293T cells. This experiment demonstrated that with the deletion of its F-box domain, cyclin F is unable to degrade Vif, thereby confirming the involvement of SCF E3 ligase machinery in cyclin F-mediated Vif degradation (Fig. 7*D*).

On confirming the proteasomal degradation of Vif by cyclin F, we performed *in vivo* ubiquitination assays of Vif in the presence of cyclin F during HIV-1 infection. TZM-bl cells were transfected with either empty vector or cyclin F and infected with 0.5 m.o.i. NL4-3 virus after 24 h of transfection. Cells were treated with MG132 for 12 h prior to harvesting at 48 hpi. The prepared lysates were used for immunoprecipitation using Vif antibody followed by immunoblotting using Lys-48 linkage-specific polyubiquitin antibody. Enhanced ubiquitin linkages were detected in cyclin F-overexpressed lysates (Fig. 7*E*), thus substantiating that cyclin F regulates HIV-1 Vif expression through polyubiquitination-mediated proteasomal degradation. Additionally, we also analyzed whether HIV-1 Vif possesses any reciprocal regulatory effect on cyclin F, as reported in the case of MDM2 E3 ligase (18). However, we did not observe any change in cyclin F expression with increasing doses of Vif (Fig. 7*F*), thus indicating that Vif may not be directly responsible for the cyclin F down-regulation observed during infection (Fig. 4*A*). Thus, overall, the above results delineate that HIV-1 NL4-3 Vif is a substrate of the SCF^cyclin F^ E3 ligase that leads to its ubiquitination and subsequent proteasomal degradation.

##### Cyclin F-mediated Vif Degradation Regulates APOBEC3G Expression to Modulate Viral Infectivity

**FIGURE 8.**
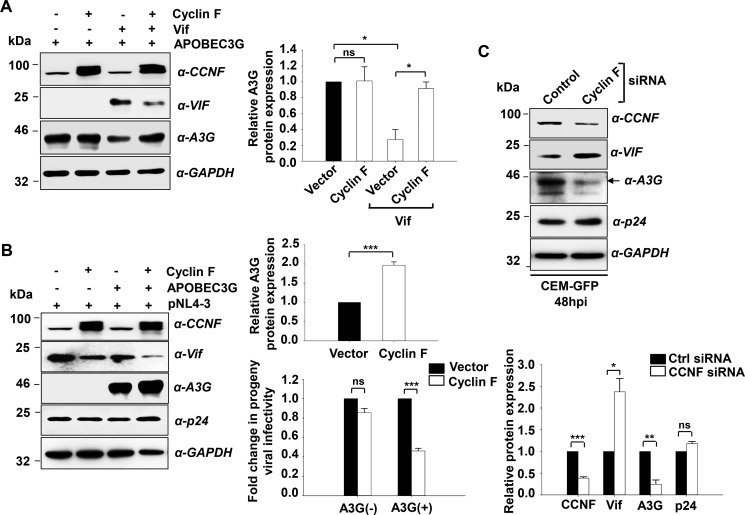
**Cyclin F regulates A3G expression during HIV-1 infection to regulate progeny viral infectivity.** *A*, cyclin F restores A3G expression to significant levels through Vif degradation. HEK293T cells were co-transfected with cyclin F and APOBEC3G in the presence or absence of the Vif expression construct, and cells were harvested 48 h post-transfection. The effect of cyclin F on A3G is not observed in the absence of Vif, demonstrating an indirect regulation of A3G by cyclin F (*left panel*). Densitometric analysis for the same is shown (*right panel*) (*n* = 3). *B*, cyclin F decreases progeny viral infectivity through Vif degradation and restoration of A3G. HEK293T cells were co-transfected with cyclin F and pNL4-3 in the presence or absence of A3G expression construct. Cells harvested 48 h post-transfection showed enhancement in A3G expression in the presence of cyclin F with simultaneous Vif degradation. Densitometric analysis for A3G expression is shown (*upper right panel*) (*n* = 3). Analysis of infectivity of the progeny virions in TZM-bl cells shows that cyclin F reduces viral infectivity in the presence of A3G (*lower right panel*). In the absence of A3G, the infectivity of progeny virions was not found to be altered with cyclin F expression (*n* = 3). *C*, silencing of endogenous cyclin F, followed by HIV-1 infection in CEM-GFP cells mimics the physiological scenario where Vif is found to be up-regulated and A3G down-regulated with no change in p24 expression. Densitometric analysis for the same is shown in the *lower panel* (*n* = 3). Data represent mean ± S.E.

HIV-1 Vif is essential for the production of infectious virions in primary cells and certain non-permissive T cell lines including H9 and CEM (41, 42), as these cells contain APOBEC3G (A3G), which interferes with viral replication and the production of infectious virions. To understand the relevance of cyclin F-mediated Vif regulation, we co-transfected pCMV4-HA-A3G along with cyclin F in the presence and absence of Vif in HEK293T cells, as A3G-transfected cells mimic a non-permissive cellular phenotype. Cells harvested at 48 h post-transfection were used for immunoblot analysis. We observed that in cells where only cyclin F and A3G were co-transfected, A3G expression remained the same as that of empty vector-transfected cells (Fig. 8*A*). Co-transfection of Vif and A3G showed degradation of A3G, in line with previous reports. However, in cells where all three constructs were co-transfected, A3G expression was found to be restored along with the degradation of Vif (Fig. 8*A*). This experiment thus indirectly implicates cyclin F in retaining cellular APOBEC3G levels by the down-regulation of Vif expression.

Further, to understand the physiologic relevance of the above observations, we co-transfected cyclin F along with pNL4-3 in the presence and absence of A3G in HEK293T cells. Cells were harvested at 48 h post-transfection, and immunoblot analysis of lysates demonstrated that cyclin F-mediated degradation of Vif leads to augmentation in the expression of A3G (Fig. 8*B*, *left* and *upper right panel*). Culture supernatants were collected and infectivity assays were performed using TZM-bl infectivity assay. We observed that the effect of cyclin F on viral infectivity was seen only in the presence of A3G in HEK293T cells (Fig. 8*B*, *lower right panel*).

As we observed earlier the regulatory effect of cyclin F on the infectivity of virions produced from CEM-GFP cells (Fig. 3), we wanted to analyze the expression of A3G in these cells. We thus performed gene silencing experiments of cyclin F in CEM-GFP cells. Cells were infected with 0.5 m.o.i. virus after 24 h of siRNA transfection and harvested at 48 hpi. Immunoblot analyses of the lysates were performed, which demonstrated that during infection, when cyclin F expression is significantly down-regulated, Vif expression is elevated ultimately leading to an attenuation of A3G expression. However, p24 expression remains unaltered here. Thus, the above experiments collectively demonstrate that cyclin F indirectly stabilizes A3G expression through the inhibition of Vif expression and thereby regulates viral infectivity.

Finally, we have identified cyclin F as a novel endogenous regulator of HIV-1 Vif that leads to its ubiquitination and proteasomal degradation. This in turn leads to the modulation of A3G expression and ultimately results in the regulation of progeny virion infectivity in non-permissive cells.

## Discussion

In the present study, we have identified cyclin F as a significantly down-regulated gene in HIV-1-infected CD4^+^ T cells. Cyclin F is the largest cyclin, with a molecular weight of nearly 100 kDa, and is ubiquitously expressed in most cell types (30). It is unique in comparison with other cyclins, as it possesses an F-box domain that is essential for the formation of SCF ubiquitin ligase through Skp1 interaction (25). Down-regulation of cyclin F expression was observed in HIV-1-infected CD4^+^ primary T cells as well as in CD4^+^ T cell lines. This prompted us to pursue cyclin F characterization and to identify its possible implication in HIV-1 infection. We report here that cyclin F is an intrinsic cellular regulator of HIV-1 Vif, which mediates proteasomal degradation of Vif through the SCF^cyclin F^ E3 ubiquitin ligase, thereby negatively regulating the infectivity of progeny virions produced from non-permissive cells.

Our preliminary overexpression and knockdown studies of cyclin F in CEM-GFP T cells implied its negative effect on viral infectivity with no significant impact on virus production, suggesting that cyclin F might be important for the infectivity of the virion. The viral infectivity factor, Vif, a 23-kDa HIV-1 accessory protein, is essential for viral replication in primary CD4^+^ T cells and certain non-permissive cells (43). Vif plays a crucial role in maintaining viral infectivity in non-permissive cells by hijacking the host ubiquitin ligase complex and mediating proteasomal degradation of APOBEC3G present in these cell types (13, 15). Vif also impairs the transcription (44) as well as translation of APOBEC3G (43, 45).

Vif is a late expressing viral protein (46, 47), and cyclin F exhibited progressive down-regulation with infection (Fig. 3*A*). The effect of cyclin F on Vif expression was therefore studied using overexpression studies. Surprisingly, here we observed that Vif was down-regulated dose-dependently with cyclin F overexpression, which confirmed that cyclin F and Vif share an inverse correlation. Knockdown studies of endogenous cyclin F further reaffirmed a negative impact of cyclin F on Vif expression.

Previous reports have shown that Vif undergoes post-translational modification by ubiquitination (48) and can undergo proteasomal degradation by Nedd4 and AIP4 (17). Apart from this, Vif, being part of the Cul5 E3 ligase, has been shown to undergo auto-ubiquitination (49). APOBEC3G polyubiquitination and subsequent degradation have been reported to be dependent also on Vif polyubiquitination by Cul5 E3 ligase (50). MDM2 E3 ligase has been reported previously as an E3 ligase of HIV-1 Vif that mediates proteasomal degradation of Vif (18). Despite all this evidence, a clear picture of Vif ubiquitination and its proteasomal degradation remains to be elucidated. Also, the regulation of Vif expression during infection has been minimally studied.

To resolve our questions regarding cyclin F and Vif, we investigated the association of both these proteins. Co-immunoprecipitation and co-localization assays were performed, which demonstrated that cyclin F interacts physically with HIV-1 Vif. Analysis of the endogenous interaction of cyclin F and Vif in infected T cells demonstrated that although cyclin F is down-regulated during infection, the residual endogenous cyclin F can still interact with Vif under physiological conditions. This implies that HIV-1 Vif could be a potential substrate of the SCF^cyclin F^ E3 ubiquitin ligase, wherein cyclin F functions as the substrate-binding protein.

The substrate specificity in case of the SCF E3 ligases is maintained by the F-box proteins that bind to the precise substrate and recruits the substrate to the ubiquitin machinery (51–53). Cyclin F is known to bind to substrate proteins containing the CY motif (R*X*L/R*X*I) through a hydrophobic patch in its cyclin domain (28, 29). Scanning of the amino acid sequence of Vif revealed the presence of a well conserved single CY motif (RKL) at amino acid position 167–169 in the C-terminal region of Vif. We analyzed the interaction of cyclin F with the CY mutant of HIV-1 Vif, which did not co-immunoprecipitate with cyclin F, suggesting that cyclin F interacts with HIV-1 NL4-3 Vif through its CY motif.

As the interaction between cyclin F and Vif was characterized, we then analyzed the involvement of proteasome machinery in the cyclin F-mediated regulation of Vif expression. Proteasomal degradation assays performed using MG132 significantly restored Vif expression even with cyclin F overexpression. We also observed enhanced Vif polyubiquitination in the presence of exogenous cyclin F with the addition of MG132. Moreover, an F-box deletion mutant of cyclin F failed to bring about any down-regulation of Vif expression.

**FIGURE 9.**
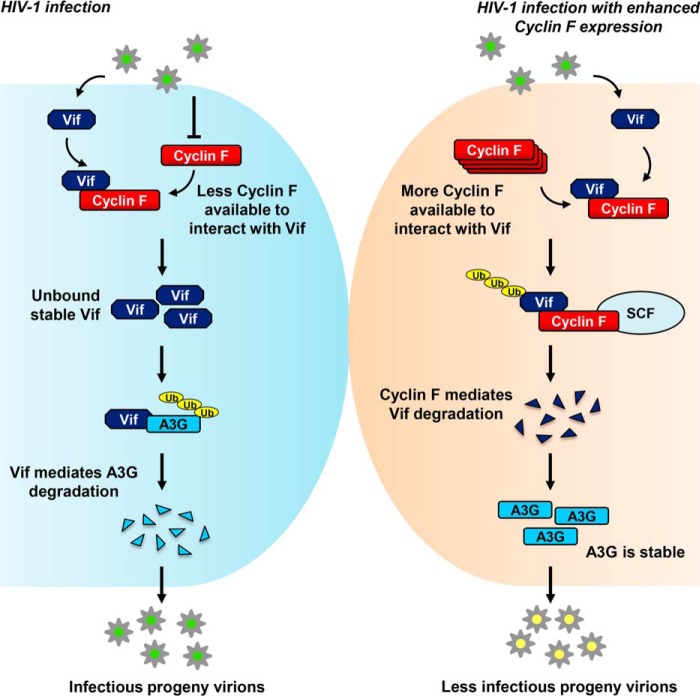
**SCF^cyclin F^ restricts HIV-1 virion infectivity by ubiquitination and proteasomal degradation of Vif and restoration of A3G levels.** *Left*, during HIV-1 infection, cyclin F undergoes down-regulation. The available cyclin F still interacts with its substrate Vif during infection. The unbound stable Vif, however mediates degradation of APOBEC3G, leading to the production of infectious virions for the next round of replication. *Right*, in the case of cyclin F overexpression and subsequent HIV-1 infection, more cyclin F is available to interact with Vif, leading to enhanced ubiquitination and proteasomal degradation of Vif through the SCF^cyclin F^ E3 ligase. This results in the stabilization of APOBEC3G, leading to the release of less infectious progeny virions.

Furthermore, to delineate the biological relevance of cyclin F-mediated Vif degradation, we analyzed the effect of this regulation on APOBEC3G expression. Interestingly, we observed that cyclin F-mediated degradation of Vif restored A3G expression, the physiologic effect of which was reflected in the regulation of viral infectivity, where less infectious viral particles were released in the presence of cyclin F and A3G. Overall, these experiments clearly demonstrated that cyclin F mediates polyubiquitination and proteasomal degradation of HIV-1 Vif through physical interaction, which regulates the infectivity of progeny virions produced from non-permissive cells through the restoration of A3G expression. (A proposed mechanistic model is depicted in Fig. 9).

Cyclin F mRNA expression is tightly regulated during the course of a normal cell cycle, which peaks in the late S and G_2_ phases and declines on entry into mitosis (30), although the precise mechanism of its regulation is unknown. As HIV-1 infection induces G_2_/M arrest, it is likely that cyclin F mRNA is down-modulated as a consequence of innate host cell cycle regulation. The biological interaction of cyclin F and Vif followed by identification of cyclin F as a negative regulator of Vif and thereby viral infectivity raises the possibility that cyclin F down-regulation during infection in CD4^+^ T cells could be a virus-mediated effect. Understanding the down-regulation of cyclin F in the context of HIV-1 infection in CD4^+^ T cells is therefore important and necessitates further studies. Also, apart from the function we have identified here, we believe that cyclin F could be involved in other cellular mechanisms in the context of HIV-1 infection, which would be interesting to explore.

Because of the explicit role that Vif plays in maintaining viral infectivity, it is a potential target for therapeutic interventions. Several approaches have been proposed to disrupt the Vif-APOBEC3G/Vif-CBF-β interaction (54, 55), including small molecule inhibitors (56, 57). Yet another novel strategy is the identification of host pathways that impede HIV-1 Vif function, as is the case with ASK1, which interferes with EloB/C binding on Vif (58), and HDAC6, which brings about autophagic degradation of Vif (59). In the present study, by identifying cyclin F as an endogenous regulator of HIV-1 Vif, we have deciphered a novel host cellular pathway for Vif regulation, and thereby viral infectivity in HIV-1-infected CD4^+^ T cells. Thus, this study could be further exploited to develop novel therapeutic strategies targeting the Vif protein of HIV-1.

## Experimental Procedures

### 

#### 

##### PBMCs, CD4^+^ T Cells, and Cell Lines

A buffy coat of seronegative blood donors was procured from the Indian Serological Institute Blood Bank, Navi Peth, Pune, India, and PBMCs were isolated using Histopaque 1077 (Sigma) by gradient centrifugation. CD4^+^ T cells were purified from PBMCs using flow cytometry-based cell sorting. Jurkat and HEK293T cell lines were obtained from the NCCS Cell Repository, Pune, India. CEM-GFP (catalog No. 3655) (60) and TZM-bl (catalog No. 8129) (61) were obtained from the NIH AIDS Reagent Program, Division of AIDS, NIAID, National Institutes of Health. HEK293T and TZM-bl cells were grown in DMEM (Invitrogen), and PBMCs, purified CD4^+^ T cells, and Jurkat and CEM-GFP cells were grown in RPMI 1640 medium (Invitrogen) containing 10% FBS and penicillin-streptomycin (Invitrogen) at 37 °C in a humidified 5% CO_2_ incubator. For CEM-GFP cells, the medium was supplemented with 500 μg/ml G418 (Invitrogen).

##### Plasmids and siRNA

pNL4-3 (catalog No. 114), a molecular clone of HIV-1, was obtained from the National Institutes of Health AIDS repository (62). pCMV-FLAG-cyclin F and its deletion mutant, pCMV-FLAG-ΔFbox-cyclin F, were cloned in our laboratory in pCMV-Tag2B mammalian expression vector (Agilent Genomics) using restriction sites XhoI and BamHI. pCMV-myc-Vif was a kind gift from Dr. A. C. Banerjea (63). The pCMV-myc-Vif (RKL/AAA) point mutant was generated using the QuikChange II site-directed mutagenesis kit according to the manufacturer's protocol (Agilent Technologies) with pCMV-myc-Vif as template. Details of the cyclin F cloning and Vif mutagenic primers are given in Table 2. pCMV4-HA-A3G (catalog No. 9951) was procured from the National Institutes of Health AIDS repository (45). Human *CCNF* siGENOME SMARTpool siRNA (M-003215-02) (GE Healthcare Dharmacon) was used for cyclin F silencing. The control siRNA used was non-targeting #1 siGENOME Control Pool (D-001206-13-20) (GE Healthcare Dharmacon). The cyclin F shRNA lentiviral constructs from Open Biosystems was a kind gift from Dr. Michael R. Green. The sequences and clone IDs of the constructs are: shRNA1, 5′-TATGGATGCTTTGTGAGTC-3′ (clone ID: V2LHS_150290); shRNA2, 5′-AGGTTTATCCGCTTCACCT-3′ (clone ID: V3LHS_322806); shRNA3, 5′-TATTCTTCGCTTTGTAGGA-3′ (clone ID: V3LHS_322803); and non-silencing shRNA, 5′-TCTCGCTTGGGCGAGAGTAAG-3′.

**TABLE 2:** **Primers used for cloning of cyclin F and ΔFbox-cyclin F and generation of Vif point mutant**

Primer	Primer sequence*^a^*
Cyclin F	F, 5′-GATATAGGATCCATGGGGAGCGG-3′
	R, 5′-CAGCTCGAGTTACAGCCTCACAA-3′
ΔFbox-cyclin F	F, 5′-GTACGGATCCTCCCAGCTTGGACTGGAGGT-3′
	R, 5′-CAGCTCGAGTTACAGCCTCACAA-3′
CY Mut Vif	F, 5′-GCCACCTTTGCCTAGTGTTGCGGCAGCGACAGAGGACAGATGGAACAAGCC-3′
	R, 5′-GGCTTGTTCCATCTGTCCTCTGTCGCTGCCGCAACACTAGGCAAAGGTGGC-3′

*^a^* F, forward; R, reverse.

##### Antibodies

The antibodies against cyclin F (rabbit, catalog No. sc-952, lot C0116; immunoblotting and immunoprecipitation), HIV-1 Vif (mouse, catalog No. sc-69731, lot F2211; immunoblotting), HIV-1 Vif (mouse, catalog No. sc-69732, lot H2907; immunoprecipitation), and GAPDH (mouse, catalog No. sc-32233, lot H2114) were procured from Santa Cruz Biotechnology. The Skp1 antibody (rabbit, catalog No. 100-401-A08, lot 15426) was procured from Rockland Immunochemicals. APOBEC3G antiserum (ApoC17, rabbit, catalog No. 10082, lot 110113) and p24 antiserum (rabbit, catalog No. 4250) were procured from the National Institutes of Health AIDS repository. Polyclonal anti-sheep Nef antibody was a kind gift from Prof. Mark Harris (64). Monoclonal anti-FLAG antibody (mouse, catalog No. F3165) was procured from Sigma.

##### Virus Stock Preparation

The HIV-1 pNL4-3 molecular clone was transfected in HEK293T cells using a CalPhos mammalian transfection kit (Clontech-Takara Bio) as per the manufacturer's instructions. Cell culture medium was collected 36 h post-medium change, clarified at 1800 × *g* for 10 min, filtered through a 0.45-μm filter, and concentrated by ultracentrifugation at 28,000 rpm for 2.5 h at 4 °C. The viral pellet was then resuspended in RPMI 1640 containing a final concentration of 50 mm HEPES. Aliquots were made and stored at −80 °C. A p24 antigen capture ELISA (Advanced Bioscience Laboratories) was used to calculate the concentration of virus in the stock.

##### HIV-1 Infection and Quantitation

PHA-activated PBMCs/CD4^+^ T cells were infected with 0.5 m.o.i. HIV-1 NL4-3 virus for 4 h at 37 °C in the presence of Polybrene (1 μg/ml) with intermittent mixing. The cells were then washed, suspended in complete medium supplemented with recombinant human IL-2 (Roche Applied Science) at 20 units/ml, and incubated until harvested. Jurkat, CEM-GFP, and TZM-bl cells were infected similarly. Culture supernatants from the infected cells were used to determine virus production by p24 antigen capture ELISA (Advanced Bioscience Laboratories).

##### Viral Infectivity Assays

For the calculation of infectivity of virus generated from overexpression/silencing experiments, culture supernatants collected from these experiments were quantified using a p24 ELISA, and equal amounts of viral p24 units were used subsequently to infect TZM-bl reporter cells at a confluency of 50–60%. Infectivity was calculated and compared using β-gal staining after fixing the cells with 0.25% glutaraldehyde (48 hpi). The infectivity of the virus stock was also calculated using the same β-gal staining method.

##### Transient Transfection

For overexpression and knockdown studies, HEK293T/TZM-bl cells were co-transfected with the indicated expression vectors or siRNA using Lipofectamine 2000 reagent (Invitrogen) according to the manufacturer's protocol followed by subsequent transfection/infection wherever indicated. Transfection in CEM-GFP cells was performed by nucleofection with Amaxa Cell Line Nucleofector kit V (Lonza) using program X-001. In all of the experiments, the cells were harvested 48 h post-transfection/infection for further analysis. All transfection experiments were normalized using empty vector control.

##### Immunoblotting, Co-immunoprecipitation, and Immunofluorescence

For immunoblotting, cells were lysed in lysis buffer (50 mm Tris-HCl, pH 7.4, 5 mm EDTA, 0.12 m NaCl, 0.5% Nonidet P-40, 0.5 mm NaF, 1 mm DTT, and 0.5 mm PMSF) supplemented with protease inhibitor mixture (Roche Applied Science). Nuclear and cytoplasmic extracts were prepared using NE-PER nuclear and cytoplasmic reagents (Thermo Scientific). Equal protein concentration was resolved on a 10–12% SDS-PAGE, and the protein was then transferred to a PVDF membrane (GE Healthcare), blocked using 5% nonfat dry milk, and probed with the indicated antibodies. All densitometric analyses of the immunoblots were performed by normalization to respective GAPDH levels. Co-immunoprecipitation assays were performed using the clarified lysates incubated with the indicated antibodies. The antigen-antibody complex was pulled down using an equal mixture of protein A- and G-agarose beads (Invitrogen) and resolved on 10–12% SDS-PAGE. Proteins transferred to a PVDF membrane were probed with the indicated antibodies. The blots were developed using the ECL Prime system (GE Healthcare). Immunofluorescence analysis was performed in infected TZM-bl cells by fixing the cells using paraformaldehyde. Cells were permeabilized using 0.1% Triton-X-100, blocked using 5% FCS, and incubated overnight with the indicated antibodies. α-Rabbit-Cy3 and α-mouse-Cy2 secondary antibodies (Chemicon) were used against the cyclin F and Vif antibodies, respectively. Cells counterstained using DAPI were acquired on an Olympus Fluoview image analyzer.

##### Quantitative Real-time PCR

Expression of genes was analyzed by quantitative real-time RT-PCR in a reaction mixture containing SYBR Green IQ Supermix (Bio-Rad) and a 10-pmol concentration of hGAPDH or gene-specific oligonucleotide primer pairs and amplified on a Realplex^4^ Mastercycler (Eppendorf, Germany). The -fold change values were calculated as: -Fold difference = 2^−ΔΔC_T_^, where ΔC_T_ = C_T_ (target) − C_T_ (GAPDH) and ΔΔC_T_ = ΔC_T_ (treated) − ΔC_T_ (control).

For the cell cycle gene expression analysis, the Human Cell Cycle RT^2^ Profiler PCR array (PAHS 020A) (SABiosciences, Qiagen) was used according to the manufacturer's protocol. Details of the primers used for gene expression analysis and PCR array data validation are given in Table 3.

**TABLE 3:** **List of primers used for quantitative real-time RT-PCR analysis and PCR array validation**

Primer	Primer sequence*^a^*
CCNF	F, 5′-AGATGTGTCAGATCCTGGGC-3′
	R, 5′-AGCTCTCACCTCCAGTCCAA-3′
ATM	F, 5′-GCTGTGAGAAAACCATGGAAG-3′
	R, 5′-AGTTTCATCTTCCGGCCTCT-3′
CDK4	F, 5′-CCGAAGTTCTTCTGCAGTCC-3′
	R, 5′-GGTCAAAGATTTTGCCCAAC-3′
CDKN3	F, 5′-CTCGGTTTATGTGCTCTTCCA-3′
	R, 5′-TTTTGACAGTTCCCCTCTGG-3′
CDKN1B	F, 5′-GGCCTCAGAAGACGTCAAAC-3′
	R, 5′-ACAGGATGTCCATTCCATGA-3′
RAD1	F, 5′-TATTCTGCAGTCAGAGGGGC-3′
	R, 5′-TGGGATAGTCAAGGTGGGAA-3′
SKP2	F, 5′-CCAGGAACTGCTCTCAAACC-3′
	R, 5′-GAAGGGAGTCCCATGAAACA-3′
CDKN1A	F, 5′-ACCGAGGCACTCAGAGGAG-3′
	R, 5′-GCCATTAGCGCATCACAGT-3′
GADD45A	F, 5′-GAGCTCCTGCTCTTGGAGAC-3′
	R, 5′-TTCCCGGCAAAAACAAATAA-3′
HERC5	F, 5′-TGTTGAAGAAGCTGCACAGG-3′
	R, 5′-CATTTTCTGAAGCGTCCACA-3′
GAPDH	F, 5′-GAAGGTGAAGGTCGGAGTC-3′
	R, 5′-GAAGATGGTGATGGGATTTC-3′
HIV-1 Vif	F, 5′-GAGTCTCCATAGAATGGAGG-3′
	R, 5′-CTGCTTGATATTCACACCTAGG-3′
HIV-1 p24	F, 5′-ATAATCCACCTATCCCAGTAGGAGAAAT-3′
	R, 5′-TTTGGTCCTTGTCTTATGTCCAGAATGC-3′

*^a^* F, forward; R, reverse.

##### Molecular Modeling and Protein-Protein Docking of Cyclin F and Vif

The amino acid sequence of cyclin F isoform 1 (cyclin F/*CCNF*) from human was retrieved from the NCBI-RefSeq (65) database (accession number NP_001752.2) in NCBI. The protein sequence was subjected to a protein-BLAST search to identify homologous proteins from the Brookhaven Protein Data Bank (PDB). Several templates were identified based on the e-value and sequence identity; however the cyclin domain structure of cyclin A protein from *Homo sapiens* (PDB ID: 4BCN, chain B), having 33% identity, was chosen to build the homology model. The template sequence of cyclin A protein and that of the target cyclin F sequence were then aligned using ClustalW (66), which showed residues 302–526 of cyclin F protein as aligned against the cyclin domain of the template structure. Subsequently, homology modeling was carried out using Modeler 9v13 (67) for the corresponding region of cyclin F protein. The outcomes of the modeled structures were ranked on the basis of an internal scoring function, and those with the least internal scores were identified and utilized for model validation by using several programs, such as PROCHECK (68), WHATIF (69), and ERRAT (70). The stereochemical qualities for predicted models were discerned using PROCHECK and by analyzing through a Ramachandran plot. The WHATIF server confirmed the average coarse packing qualities and non-bonded interactions among different atoms of the models; these data were validated using ERRAT. The crystal structure of Vif was obtained from the Protein Data Bank (PDB ID: 4N9F), and chain B of Vif protein (176 residues long) was used for the docking study. Docking studies were performed between the known Vif structure and the modeled structure of cyclin F protein with PatchDock server (71). PatchDock provided results that were ranked according to the geometric shape complementarity score after molecular shape representation and surface patch matching. PISA (Protein Interfaces, Surfaces, and Assemblies) was used to analyze the protein-protein interactions and binding interface of the cyclin F-Vif-docked complex (72).

##### Ubiquitination Assays

Endogenous ubiquitination assays were performed by treating the cells with proteasomal inhibitor MG132 (5 μm) for 12 h prior to harvesting. The prepared lysates were immunoprecipitated using HIV-1 Vif antibody (sc-69732) followed by immunoblotting using Lys-48 linkage-specific polyubiquitin antibody (rabbit, catalog No. 8081S, lot 2) (Cell Signaling).

##### Statistical Analysis

Statistical analysis of the experimental data was performed using Student's *t* test. Error bars represent mean ± S.E. The levels of significance shown in Figs. 1–4 and 6–8 are as follows: not significant (*ns*) ≥ 0.05; *, *p* ≤ 0.05; **, *p* ≤ 0.01; and ***, *p* ≤ 0.001.

## Author Contributions

T. A., D. M., M. K. S., and P. C. contributed to the concept of the paper. T. A., M. K. S., and D. M. designed the experiments. T. A., K. G., S. I., and P. G. performed the experiments. T. A., P. C., P. G., and D. M. wrote the paper.

## Supplementary Material

Supplemental Data

## Footnotes

2 The abbreviations used are:

Vif: viral infectivity factor
CDK: cyclin-dependent kinase
PBMC: peripheral blood mononuclear cell
m.o.i.: multiplicity of infection
hpi: hours post-infection
co-IP: co-immunoprecipitation
A3G: APOBEC3G
PHA: phytohemagglutinin
PDB: Protein Data Bank.
