# Supplementary material for: Cyclin F/FBXO1 Interacts with HIV-1 Viral Infectivity Factor (Vif) and Restricts Progeny Virion Infectivity by Ubiquitination and Proteasomal Degradation of Vif Protein through SCFcyclin F E3 Ligase Machinery
Source: J Biol Chem. 2017 Feb 9;292(13):5349–63. doi: 10.1074/jbc.M116.765842 (PMC5392680; doi:10.1074/jbc.M116.765842)
Supplement: Supplemental Data [file supp_292_13_5349__index.html]

Cyclin F/FBXO1 interacts with HIV-1 Vif and restricts progeny virion infectivity by ubiquitination and proteasomal degradation of Vif through SCF Cyclin F E3 ligase machinery — Cyclin F/FBXO1 Interacts with HIV-1 Viral Infectivity Factor (Vif) and Restricts Progeny Virion Infectivity by Ubiquitination and Proteasomal Degradation of Vif Protein through SCFcyclin F E3 Ligase Machinery — SCFcyclin F E3 Ligase Proteasomally Degrades HIV-1 Vif — Supplemental Data 

# Cyclin F/FBXO1 Interacts with HIV-1 Viral Infectivity Factor (Vif) and Restricts Progeny Virion Infectivity by Ubiquitination and Proteasomal Degradation of Vif Protein through SCFcyclin F E3 Ligase Machinery

## Supplemental Data

- Supplementary Table-1 (.pdf, 73 KB) - Array data
