# Supplementary material for: Cyclin F/FBXO1 Interacts with HIV-1 Viral Infectivity Factor (Vif) and Restricts Progeny Virion Infectivity by Ubiquitination and Proteasomal Degradation of Vif Protein through SCFcyclin F E3 Ligase Machinery
Source: J Biol Chem. 2017 Feb 9;292(13):5349–63. doi: 10.1074/jbc.M116.765842 (PMC5392680; doi:10.1074/jbc.M116.765842)
Supplement: Supplemental Data [file 10.1074_M116.765842_jbc.M116.765842-1.pdf]

Cyclin F/FBXO1 interacts with HIV-1 Vif and restricts progeny virion infectivity by ubiquitination and proteasomal degradation of Vif through SCF$^{Cyclin\ F}$ E3 ligase machinery

Tracy Augustine[1], Priyanka Chaudhary[1], Kailash Gupta[1], Sehbanul Islam[1], Payel Ghosh[2], Manas Kumar Santra[1] and Debashis Mitra[1]*

[1]National Centre for Cell Science, Pune, Maharashtra, 411007, India
[2] Bioinformatics Centre, Savitribai Phule Pune University, Pune, Maharashtra, 411007, India

Running Title: SCF$^{Cyclin\ F}$ E3 ligase proteasomally degrades HIV-1 Vif

*To whom correspondence should be addressed: Dr. Debashis Mitra, Laboratory for HIV Research, National Centre for Cell Science, Pune, Maharashtra, 411007, India

Telephone: 91-20-25708151; Fax: 91-20-25692259;
E-mail: dmitra@nccs.res.in, dmitra01@yahoo.co.in

Keywords: Human Immunodeficiency Virus (HIV), Host-Pathogen Interaction, E3 Ubiquitin Ligase, Cyclin F, F-box protein, Vif, APOBEC3G, Protein degradation, Ubiquitination, Virion infectivity

Supplemental Table 1: List of differentially modulated genes in the cell cycle PCR array

Gene	***NM_ID***	***Gene name***	***Cell cycle phase***	***Fold change***
ANAPC2	NM_013366	Anaphase promoting complex subunit 2	G1,G1-S,G2,G2-M	**-2.0849**
ATM	NM_000051	Ataxia telangiectasia mutated	Cell cycle checkpoint, cell cycle arrest and regulation	**-2.6574**
BCCIP	NM_016567	BRCA2 and CDKN1A interacting protein	G2,G2-M and cell cycle regulation	**-2.0139**
BRCA1	NM_007294	Breast cancer 1, early onset	Cell cycle checkpoint, cell cycle arrest and regulation	**-2.2346**
CCNB1	NM_031966	Cyclin B1	G2,G2-M and cell cycle regulation	**-3.5064**
CCNB2	NM_004701	Cyclin B2	M and cell cycle regulation	**-2.5491**
CCNE1	NM_001238	Cyclin E1	G1,G1-S and cell cycle regulation	**-2.4794**
CCNF	NM_001761	Cyclin F	M and cell cycle regulation	**-5.5022**
CCNG2	NM_004354	Cyclin G2	Cell cycle check point and cell cycle arrest	**-2.4794**
CCNT1	NM_001240	Cyclin T1	G2,G2-M and cell cycle regulation	**-2.2974**
CDC16	NM_003903	Cell division cycle 16 homolog (*S. cerevisiae*)	Cell cycle regulation	**-2.1287**
CDC20	NM_001255	Cell division cycle 20 homolog (*S. cerevisiae*)	M and cell cycle regulation	**-3.5554**
CDC34	NM_004359	Cell division cycle 34 homolog (*S. cerevisiae*)	G1,G1-S, cell cycle checkpoint and cell cycle arrest	**-2.4284**
CDK4	NM_000075	Cyclin-dependent kinase 4	G1,G1-S and cell cycle regulation	**-2.2038**
CDK6	NM_001259	Cyclin-dependent kinase 6	G1,G1-S and cell cycle regulation	**-2.395**
CDKN1A	NM_000389	Cyclin-dependent kinase inhibitor 1A (p21, Cip1)	Cell cycle checkpoint, cell cycle arrest and regulation	**5.278**
CDKN1B	NM_004064	Cyclin-dependent kinase inhibitor 1B (p27, Kip1)	G1,G1-S, cell cycle checkpoint, cell cycle arrest and regulation	**-2.1287**
CDKN2A	NM_000077	Cyclin-dependent kinase inhibitor 2A (melanoma, p16, inhibits CDK4)	Cell cycle check point and cell cycle arrest	**-2.8679**
CDKN2B	NM_004936	Cyclin-dependent kinase inhibitor 2B (p15, inhibits CDK4)	Cell cycle checkpoint, cell cycle arrest and regulation	**-2.1735**
CDKN3	NM_005192	Cyclin-dependent kinase inhibitor 3	G1,G1-S,G2,G2-M, cell cycle check point and cell cycle arrest	**-2.1435**
CKS2	NM_001827	CDC28 protein kinase regulatory subunit 2	G2 and G2-M	**-2.0562**
CUL3	NM_003590	Cullin 3	G1, G1-S, cell cycle check point and cell cycle arrest	**-5.4264**
DNM2	NM_004945	Dynamin 2	G2, G2-M	**-3.1383**
GADD45A	NM_001924	Growth arrest and DNA-damage-inducible, alpha	Cell cycle checkpoint, cell cycle arrest and regulation	**3.2716**

GTSE1	NM_016426	G-2 and S-phase expressed 1	G2,G2-M	**-2.395**
HERC5	NM_016323	Hect domain and RLD 5	G2,G2-M	**20.6776**
HUS1	NM_004507	HUS1 checkpoint homolog (*S. pombe*)	Cell cycle check point and cell cycle arrest	**-2.4623**
KNTC1	NM_014708	Kinetochore associated 1	Cell cycle check point, cell cycle arrest and regulation	**-2.2501**
KPNA2	NM_002266	Karyopherin alpha 2 (RAG cohort 1, importin alpha 1)	G2,G2-M	**-2.8284**
MAD2L1	NM_002358	MAD2 mitotic arrest deficient-like 1 (yeast)	Cell cycle check point and cell cycle arrest	**-2.0994**
MCM2	NM_004526	Minichromosome maintenance complex component 2	S and DNA replication	**-2.3784**
MCM3	NM_002388	Minichromosome maintenance complex component 3	S and DNA replication	**-2.2658**
MCM4	NM_005914	Minichromosome maintenance complex component 4	S and DNA replication	**-2.8089**
MCM5	NM_006739	Minichromosome maintenance complex component 5	S and DNA replication	**-2.514**
MKI67	NM_002417	Antigen identified by monoclonal antibody Ki-67	Cell cycle regulation	**-3.6553**
MRE11A	NM_005590	MRE11 meiotic recombination 11 homolog A (*S. cerevisiae*)	M	**-3.7064**
RAD1	NM_002853	RAD1 homolog (*S. pombe*)	Cell cycle check point and cell cycle arrest	**-2.5315**
RBL1	NM_002895	Retinoblastoma-like 1 (p107)	Cell cycle regulation	**-2.3784**
SKP2	NM_005983	S-phase kinase-associated protein 2 (p45)	G1,G1-S and cell cycle regulation	**-2.7702**
TFDP1	NM_007111	Transcription factor Dp-1	Cell cycle regulation	**-3.1602**
TP53	NM_000546	Tumor protein p53	Cell cycle regulation	**-3.6301**

Supplemental Table 1: List of cell cycle associated genes that were modulated in primary CD4$^+$ T cells with HIV-1 infection. Among the 84 genes in the PCR array, the table lists 41 genes which exhibited modulation of more than 2-fold with HIV-1 infection. The complete list of genes in the array is available at http://www.sabiosciences.com/rt_pcr_product/HTML/PAHS-020Z.html. (There are few changes in the gene list as compared to PAHS-020A, which has been used in the present work).
